# β-*N*-Acetylglucosaminidase *Mth*NAG from *Myceliophthora thermophila* C1, a thermostable enzyme for production of *N*-acetylglucosamine from chitin

**DOI:** 10.1007/s00253-018-9166-3

**Published:** 2018-06-25

**Authors:** Malgorzata Krolicka, Sandra W. A. Hinz, Martijn J. Koetsier, Gerrit Eggink, Lambertus A. M. van den Broek, Carmen G. Boeriu

**Affiliations:** 1 Department of Bioprocess Engineering, Wageningen University, Wageningen, The Netherlands; 2 Wageningen Food & Biobased Research, Wageningen, The Netherlands; 3 DuPont Industrial Biosciences, Wageningen, The Netherlands

**Keywords:** *Myceliophthora thermophila* C1, β-*N*-Acetylglucosaminidase, *N*-Acetylglucosamine, Chitin, Chitosan

## Abstract

Thermostable enzymes are a promising alternative for chemical catalysts currently used for the production of *N*-acetylglucosamine (GlcNAc) from chitin. In this study, a novel thermostable β-*N*-acetylglucosaminidase *Mth*NAG was cloned and purified from the thermophilic fungus *Myceliophthora thermophila* C1. *Mth*NAG is a protein with a molecular weight of 71 kDa as determined with MALDI-TOF-MS. *Mth*NAG has the highest activity at 50 °C and pH 4.5. The enzyme shows high thermostability above the optimum temperature: at 55 °C (144 h, 75% activity), 60 °C (48 h, 85% activity; half-life 82 h), and 70 °C (24 h, 33% activity; half-life 18 h). *Mth*NAG releases GlcNAc from chitin oligosaccharides (GlcNAc)_2–5_, *p*-nitrophenol derivatives of chitin oligosaccharides (GlcNAc)_1–3_-*p*NP, and the polymeric substrates swollen chitin and soluble chitosan. The highest activity was detected towards (GlcNAc)_2_. *Mth*NAG released GlcNAc from the non-reducing end of the substrate. We found that *Mth*NAG and Chitinase Chi1 from *M. thermophila* C1 synergistically degraded swollen chitin and released GlcNAc in concentration of approximately 130 times higher than when only *Mth*NAG was used. Therefore, chitinase Chi1 and *Mth*NAG have great potential in the industrial production of GlcNAc.

## Introduction

Large amounts of polysaccharides present in nature are an excellent source of valuable sugars, and chitin is one of them. Chitin is a vastly abundant polysaccharide and is used for the production of chitosan, chitin oligosaccharides, and GlcNAc (Kardas et al. 2012). Generally, chitin consists of linearly β-(1–4) linked *N*-acetylglucosamine molecules (GlcNAc). Chitosan, the deacetylated derivative of chitin, has wide application in many fields including medicine, pharmacology, environmental protection, and biobased packaging (Elieh-Ali-Komi and Hamblin 2016; Kardas et al. 2012; Van den Broek et al. 2015). Chitin oligosaccharides can be used as antimicrobial (No et al. 2002), antitumor, and anti-inflammatory agents (Azuma et al. 2015). GlcNAc has gained great attention as a candidate for multiple applications (Chen et al. 2010). In medicine, GlcNAc is considered as an inexpensive and non-toxic treatment for numerous diseases including osteoarthritis, inflammatory bowel disease, viral or bacterial infections, intestinal diseases, and cancer and for proliferation of skin cells during wound healing (Dalirfardouei et al. 2016; Salvatore et al. 2000; Xu et al. 2006; Xu et al. 2007a, b; Minami and Okamoto 2007). In cosmetics, GlcNAc is a valuable ingredient for improving skin quality (Riordan 1999; Bissett et al. 2007). In the food industry, GlcNAc is used as an additive in beer, milk, and wine (Xu et al. 2004a, b, c). Recently, GlcNAc was proposed as a biological C6 source for bioethanol production through fermentation. It was reported that fermentation with *Mucor circinelloides* NBRC6746 and *Mucor ambiguous* NBRC8092 yielded approximately 18.6 and 16.9 g/L ethanol from 50 g/L GlcNAc, respectively (Inokuma et al. 2013).

Chitin is used for industrial production of GlcNAc and is obtained mainly from exoskeletons of crustaceans and to a lesser extent from insects and fungal cell walls (Rinaudo 2006). Currently, degradation of chitin to GlcNAc from crustaceans is conducted with 15–36% HCl at 40–80 °C (Chen et al. 2010). Another production process involves the use of concentrated HCl at boiling temperature. Such harsh conditions lead to degradation of chitin and deacetylation of the monomer to glucosamine (GlcN). The produced GlcN is subsequently *N*-acetylated with acetic anhydride. However, there appears to be several problems in producing GlcNAc by chemical depolymerization of chitin, including high operational costs, low yield (below 65%), and acidic waste created by the use of HCl and acetic anhydride (Chen et al. 2010). A promising alternative for the chemical process is chitin depolymerization with enzymes. In comparison to the chemical process, enzymatic depolymerization is conducted under milder conditions and lower temperatures, and no hazardous wastes are released. In nature, chitin is degraded by three enzymes acting in concert: lytic polysaccharide monooxygenase (LPMO), chitinase, and β-*N*-acetylglucosaminidase (NAGase). LPMOs (EC 1.14.99.53) act in an oxidative way on the surface of crystalline chitin, where they introduce chain breaks and generate oxidized chain ends, thus promoting further degradation by chitinases (Vaaje-Kolstad et al. 2010). Chitinases (EC 3.2.1.14) catalyze the cleavage of the glycosidic bond in chitin chains and release chitin oligosaccharides and chitin dimers as end products. Products released by chitinase are finally converted to GlcNAc by NAGases (Ike et al. 2005; Hartl et al. 2012). According to the carbohydrate active enzymes classification (CAZy) (http://www.cazy.org/), LPMOs are classified to the auxiliary activity (AA) family AA10 and AA11, chitinases to glycoside hydrolase (GH) family 18, and NAGases to GH20 and GH3. Chitin-degrading enzymes are naturally produced by fungi, bacteria, plants, yeasts, insects, and even vertebrates, among various organisms (Bhattacharya et al. 2007; Keyhani and Roseman 1996). However, enzymes potentially appropriate for GlcNAc production from chitin at industrial scale are thermostable enzymes obtained from fungi (Østergaard and Sejr Olsen 2010). Fungal thermostable enzymes are currently used in industry, e.g., α-amylase in baking, because they tolerate high temperature, use shorter times to complete conversion, and have a long shelf life (Kristjansson 1989; Østergaard and Sejr Olsen 2010). Fungal thermophilic NAGases with a temperature optimum of 50–65 °C have been characterized from fungi, i.e., *Aspergillus niger* (Pera et al. 1997), *Beauvaria bassiana* (Bidochka et al. 1993), *Lentinula edodes* (Konno et al. 2012), *Trichoderma harzianum* (Ulhoa and Peberdy 1991; Lorito et al. 1994; Lisboa De Marco et al. 2004; Koga et al. 1991), and *Penicillium oxalicum* (Ryslava et al. 2011)*.* However, after prolonged incubations at optimal or higher temperatures, their enzymatic activity diminished drastically or was lost. Therefore, there is a need for more robust thermophilic NAGases with improved thermostability. Recently, we have reported the production and the characterization of the thermostable chitinase Chi1, an endochitinase from the thermophilic filamentous fungus *Myceliophthora thermophila* C1 (Krolicka et al. 2018). In this study, we present the cloning and the properties of NAGase *Mth*NAG, a second enzyme in the chitinolytic machinery of *M. thermophila* C1.

## Materials and methods

### Substrates and chemicals

Chitin from shrimp shells, Schiff’s reagent, 4-nitrophenyl-*N*-acetylglucosamine (GlcNAc-*p*NP), 4-nitrophenyl-*N*,*N′*-diacetyl-*β*-D-chitobiose ((GlcNAc)_2_-*p*NP) and 4-nitrophenyl-*β*-D-*N*,*N′*,*N″*-triacetylchitotriose ((GlcNAc)_3_-*p*NP), and 4-nitrophenyl-*N*-acetylgalactosamine (GalNAc-*p*NP) were obtained from Sigma-Aldrich (St. Louis, USA). GlcNac was obtained from Sigma-Aldrich (St. Louis, USA). Chitin oligosaccharides (GlcNAc)_2–6_ were obtained from Megazyme (Co. Wicklow, Ireland). Chitosan (90% deacetylation degree (% DDA), 100 kDa molecular weight) was purchased from Nippon Suisan Kaisha LTD. Swollen chitin was prepared according to Monreal and Reese (1969) with some modifications as described by Krolicka et al. (2018). Swollen chitin had a moisture content of 95.7%. All other chemicals were of the highest purity available. Chitinase Chi1 from *M. thermophila* C1 was isolated and purified as described by Krolicka et al. (2018).

### Sequence analysis of MthNAG

The nucleotide sequence of the gene encoding for *Mth*NAG (*Mthnag*) and deduced amino acid sequence of *Mth*NAG were analyzed using Clone Manager software. BLAST analysis was performed at the NCBI server (https://blast.ncbi.nlm.nih.gov/Blast.cgi). Conserved domains were detected with Conserved Domain Search and Conserved Domain Database (Marchlerbauer et al. 2015) at the NCBI server (https://www.ncbi.nlm.nih.gov/Structure/cdd/wrpsb.cgi). The signal peptide was analyzed at the SignalP 4.0 server (http://www.cbs.dtu.dk/services/SignalP/), and the theoretical isoelectric point (p*I*) was calculated with the Compute pI/Mw tool on ExPASy server (https://web.expasy.org/compute_pi/). Potential *N*-linked and *O*-linked glycosylation sites were predicted by NetOGlyc 4.0 Server and NetNGlyc 1.0 Server (http://www.cbs.dtu.dk/services/). Prediction of the protein secondary structure and 3D was performed with the Phyre2 web portal (www.sbg.bio.ic.ac.uk/phyre2). 3D modeling was performed on the basis of the crystal structure of insect β-*N*-acetyl D-hexosaminidase of hex12 hexosaminidase (template ID: c3nsnA) which showed 90% coverage with the *Mth*NAG sequence.

### Fungal strain

The ascomycetous fungus *Myceliophthora thermophila* C1 is listed in the All-Russian Collection of Microorganisms of the Russian Academy of Sciences with accession number VKM F-3500D.

### Gene cloning and protein production

The genome of *M. thermophila* C1 was screened for genes encoding for NAGases using a protein sequence of the fungal GH family 20 (XP_003656648) published at NCBI. This sequence was blasted against the *M. thermophila* C1 genome data base (Genencor International B.V., a DuPont company), and only one gene sequence of a putative GH20 NAGase was found in the *M. thermophila* C1 genome data base and this gene sequence has previously been published in a patent (Verdoes et al. 2010). The native gene encoding for a putative GH20 NAGase found in the genome of *M. thermophila* C1 was designated *Mthnag*. *Mthnag* was chosen for overexpression and characterization. The gene *Mthnag* was amplified from genomic *M. thermophila* C1 DNA with phusion DNA polymerase using the following designed primers (forward: GCTCGATTAAACATGTGGTCGCCG, reverse: GATGCGACCCGAATTCTCAAGCGACGA). The PCR program was as follows: 1× 98 °C for 30 s, 35× (1× 98 °C for 10 s, 35× 63.8 °C for 30 s, 72 °C for 30 s), 1× 72 °C for 10 min. The amplified gene was cloned into a C1 expression vector and homologously overexpressed in *M. thermophila* C1 according to the method described by Visser et al. (2011). In short, the expression cassette, containing the chi1 promoter, the gene *Mthnag*, and the terminator obtained from the expression vector, was transformed into a low-protease/(hemi-) cellulase-free *M. thermophila* C1-expression host. Ninety-six randomly integrated transformants were grown in a microtiter plate (Verdoes et al. 2007) and screened for NAGase activity in the culture broth using GlcNAc-*p*NP as substrate. The transformant showing the highest activity of *Mth*NAG was selected for a 2-L fed-batch fermentation as previously reported (Visser et al. 2011). The strain was grown aerobically in mineral medium, with glucose as carbon source, ammonium sulfate as nitrogen source, and trace elements for essential salts (Verdoes et al. 2010). *Mth*NAG was produced at pH 6.0 and 32 °C. The broth containing *Mth*NAG was centrifuged at 20,000×g for 20 min, and the supernatant was filtered to remove cell biomass (filter 0.45 μm, Sartorius), concentrated fourfold (5 kDa PES membrane, Vivace 70, Sartorius), dialyzed against 10 mM potassium phosphate buffer pH 6.0, and freeze-dried to obtain a crude enzyme preparation.

### Purification of *Mth*NAG

The freeze-dried crude enzyme preparation (0.5 g) was dissolved in 50 mL 0.05 M Bis-Tris buffer pH 7.0 (buffer A) and purified by anion-exchange chromatography (IEX) and size exclusion chromatography (SEC) on an ÄKTA™ pure system. For IEX, the 50-mL sample was loaded onto a HiPrep DEAE FF 16/10 column (GE Healthcare Bio-Science AB, Uppsala, Sweden) and proteins were eluted with a gradient elution of 1 M NaCl in buffer A as follows: 0–20% for 10 column volumes (CV) and 20–45% for 10 CV with a flow rate of 5 mL min^−1^. Fractions were collected and screened for NAGase activity. The fraction with the highest NAGase activity was loaded onto a HiLoad 16/600 Superdex 75 pg SEC column (GE Healthcare Bio-Science AB, Uppsala, Sweden). Proteins were eluted isocratic with buffer A containing 0.15 M NaCl with a flow rate of 0.5 mL min^−1^. The absorbance was measured at 280 nm. Protein concentration was determined using the bicinchoninic acid assay (BCA) according to the instructions of the supplier (Pierce) with bovine serum albumin as standard. The evaluation of the purity and molecular weight of purified *Mth*NAG was performed by matrix-assisted laser desorption time-of-flight mass spectrometry (MALDI-TOF-MS) as described by Krolicka et al. (2018).

### Enzyme activity assays and kinetic parameters

For the standard enzyme assay, the enzyme solution (0.01 mL) was incubated with GlcNAc-*p*NP (2 mM) in 0.09 mL citrate-phosphate buffer (0.1 M, pH 4) at 50 °C in a microtiter plate. After 10 min incubation, 0.2 mL Tris/HCl buffer (0.25 M, pH 8.8) was added and the absorbance of released *p*-nitrophenol (*p*NP) was measured at 405 nm using a Tecan Safire plate reader (Grodig, Austria). One enzyme unit (U) was defined as the amount of enzyme required to release 1 μmol of *p*NP per minute. Activity on chitin oligosaccharide derivatives (*p*NP derivatives) was assayed with (GlcNAc)_1–3_-*p*NP at standard enzyme conditions. Activity for chitin and chitosan was assayed with 0.8 μM *Mth*NAG incubated with 0.45% (*w*/*v*) swollen chitin or soluble chitosan in 0.96 mL Na-acetate buffer (0.05 M, pH 4.5) at 50 °C for 10 min. Reactions were terminated by heating at 96 °C for 10 min and subsequently centrifuged at 20,000×g for 5 min. The reducing sugars released from chitosan were analyzed with the *p*-hydroxybenzoic acid hydrazide (PAHBAH) assay (Lever 1973). For chitin, released GlcNAc was determined by high-performance anion-exchange chromatography (HPAEC). One U was defined as the amount of enzyme that liberated 1 μmol GlcNAc per minute from chitin or chitosan. The *K*_m_, *V*_max_, *k*_cat_, and *K*_i_ of *Mth*NAG were determined for (GlcNAc)_2_ and GlcNAc-*p*NP. For GlcNAc-*p*NP, 2.1 nM *Mth*NAG was incubated with GlcNAc-*p*NP in the range of 0.05–4 mM under standard enzyme assay conditions. For (GlcNAc)_2_, 2.1 nM *Mth*NAG was incubated with (GlcNAc)_2_ in the range of 0.05–3.5 mM in citrate-phosphate buffer (0.1 M, pH 4.5) and released GlcNAc was determined by HPAEC. The kinetic parameters were calculated with GraphPad Prism software v.7.0 (GraphPad Software Inc., San Diego, CA). For all experiments, each reported value was the average of duplicate tests.

### Biochemical characterization

The influence of pH on the activity of *Mth*NAG was determined by incubating 2.1 nM *Mth*NAG at different pH levels (3.0–7.0) in citrate-phosphate buffer (0.1 M) using the standard enzyme assay conditions. The influence of temperature on the activity of *Mth*NAG was analyzed by incubating 2.1 nM *Mth*NAG at the temperature range of 30–80 °C using the standard enzyme assay conditions. Thermostability was determined by pre-incubating 2.1 nM *Mth*NAG at various temperatures (50–70 °C) in citrate-phosphate buffer (0.1 M, pH 4.5) for different time intervals, and the remaining enzyme activity was determined by performing the standard enzyme assay. For all experiments, each reported value was the average of duplicate tests.

### Electrophoresis and identification of glycosylated proteins

Sodium dodecyl sulfate-polyacrylamide (10% (*w*/*v*)) gel electrophoresis (SDS-PAGE) was performed under reducing conditions using a NuPAGE Novex System (ThermoFisher Scientific, Bleiswijk, The Netherlands) with 10% (*w*/*v*) Bis-Tris gels. Gels were stained with SimplyBlue™ SafeStain according to the recommendation of the supplier (ThermoFisher Scientific). The isoelectric point of the enzyme was estimated by isoelectric focusing (IEF) using PhastGel™ IEF on a Pharmacia LKB Phast System (Pharmacia Biotech, Uppsala, Sweden) with a broad protein calibration kit (pH 3–10, GE Healthcare) as standard. Proteins were stained with Coomassie blue R-2. Glycosylation of proteins was detected by staining the SDS-PAGE gels with periodic acid-Schiff staining (PAS) (Zacharius et al. 1969).

### Time course of hydrolysis of chitin oligosaccharides and *p*NP derivatives

To analyze the mode of action, the hydrolysis of chitin oligosaccharides (GlcNAc)_2–5_ and (GlcNac)_2–3_-*p*NP (0.18 mM) was performed with 2.1 nM *Mth*NAG in 0.33 mL citrate-phosphate buffer (0.1 M, pH 4.5) at 50 °C. Time-point samples were taken, heated at 96 °C for 5 min, and centrifuged at 20,000×g for 5 min. Hydrolysis products were analyzed by HPAEC and each reported value was the average of duplicate tests. The quantification was based on calibration curves prepared for each chitin oligosaccharide.

### Synergistic effect of *Mth*NAG and Chitinase Chi1 on chitin hydrolysis

To study the synergistic effect of *Mth*NAG and Chitinase Chi1, chitin hydrolysis was performed with 0.5% (*w*/*v*) swollen chitin in 1 mL citrate-phosphate buffer (0.1 M, pH 5.0) in two parallel runs: run I containing 0.8 μM *Mth*NAG and run II containing 0.8 μM *Mth*NAG and 1.2 μM Chitinase Chi1. Samples were incubated at 50 °C with agitation at 800 rpm. In time, samples were taken, heated at 96 °C for 10 min, and centrifuged at 20,000×g for 5 min. Hydrolysis products were analyzed by HPAEC, and each reported value was the average of the duplicate test. The quantification was based on the calibration curve for GlcNAc. GlcNAc production yields were calculated by comparing the amount of GlcNAc released to the maximal theoretical yield, which is equal to the initial substrate concentration considering that 1 mg of dry chitin could produce a maximum of 1.09 mg of GlcNAc.

### HPAEC

HPAEC was performed on an ICS-3000 ion chromatography HPLC system equipped with a CarboPac PA-1 column (2 × 250 mm) in combination with a CarboPac PA-guard column (2 × 25 mm) at 22 °C and a pulsed electrochemical detector (PAD) in pulsed amperometric detection mode (Dionex) at 30 °C. The column was equilibrated with water. Sugars were eluted at a flow rate of 0.25 mL min^−1^. The gradient used was 0–25 min H_2_O, 25–65 min at 0–0.045 M NaOH, 65–70 min at 0.045 M NaOH–1 M sodium acetate in 0.1 M NaOH, 70–75 min at 1 M sodium acetate in 0.1 M NaOH, 75–75.1 min 1 M sodium acetate in 0.1 M NaOH–0.1 M NaOH, 75.1–80 min 0.1 M NaOH, and 80–95 min H_2_O. The PAD signal was increased by post column addition of 0.5 M NaOH at a flow rate of 0.15 mL min^−1^.

## Results

### Sequence analysis of *Mth*NAG

**Fig. 1:**
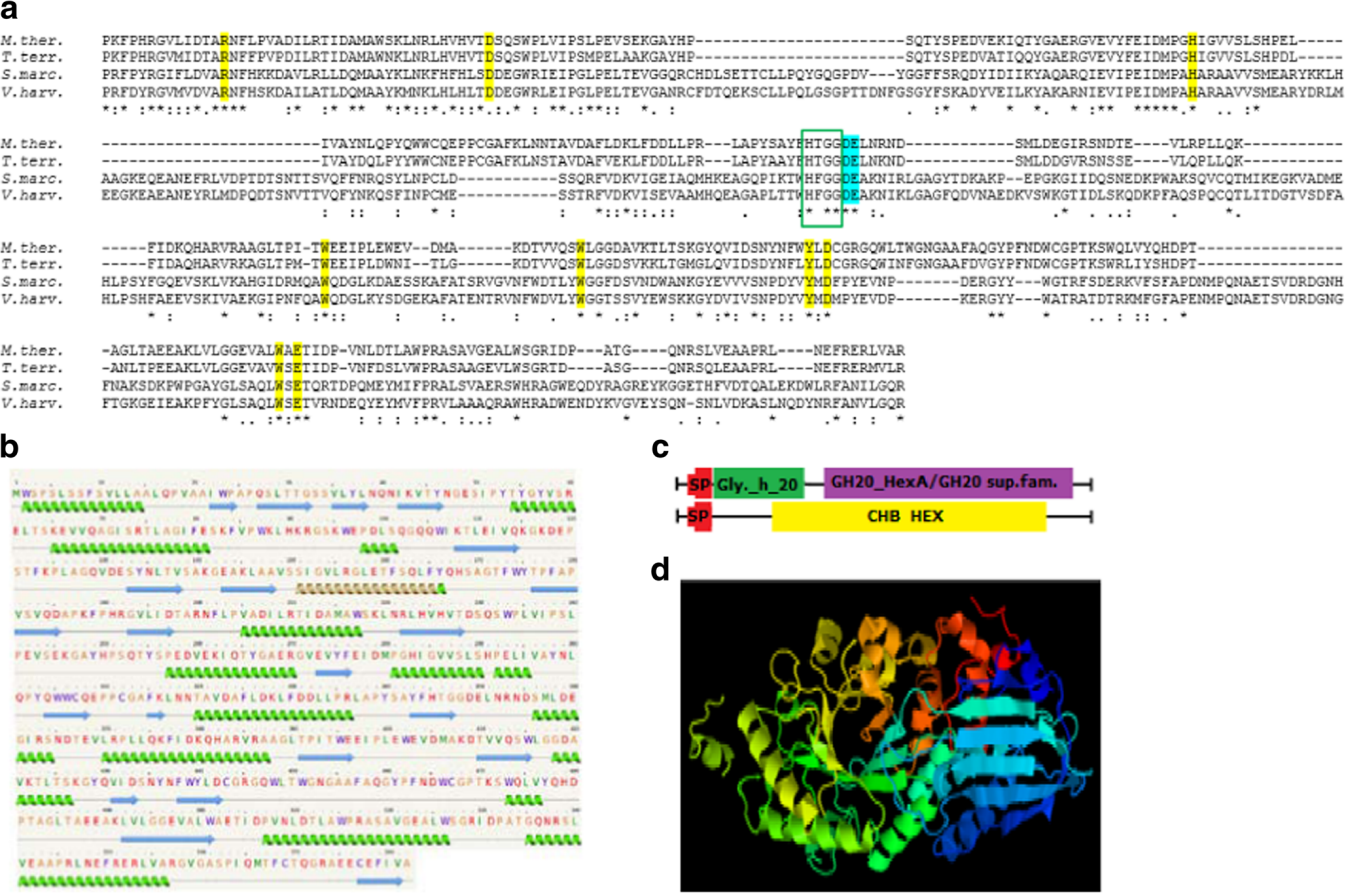
Multiple amino acid sequence alignment of the active sites of selected GH family 20 hexosaminidases (**a**). The sequence alignment was conducted with Clustal Omega. The deduced amino acid sequence of *Mth*NAG from *Myceliophthora thermophila* C1 (*M.ther.*) was aligned with GH family 20 proteins from *Thelavia terrestris* (*T.terr*.; GenBank: XP_003656648), chitobiase from *Serratia marcescens* (*S.marc*.; Swiss-Prot: Q54468), and chitobiase from *Vibrio harveyi* (*V.harv*.; Swiss-Prot: P13670). The conserved HXGG motif is marked with a green box. The conserved aspartate and catalytic glutamate are marked in blue. An asterisk (*) indicates highly conserved residues; double (:) and single (.) dots indicate conserved similar residues. The conserved amino acids in GH20_HexA_HexB-like domain (accession: cd06562) are marked in yellow. Secondary structure (**b**) was analyzed with the Phyre2 web portal. The conserved domains GH20_HexA_HexB-like domain (accession: cd06562), Glyco_hydro_20 domain (accession: pfam00728), and CHB_HEX domain (accession: PF03173) were identified with BLAST and the signal peptide (SP) was predicted with the SignalP 4.0 server (**c**). 3D modeling of *Mth*NAG was performed with Phyre2 web portal (**d**)

The putative gene (*Mthnag*) encoding for NAGase *Mth*NAG has an ORF of 2005 bp. The deduced 582-amino acid sequence of *Mth*NAG was predicted to have a molecular weight of 62.6 kDa, a theoretical p*I* of 5.20, an *N*-terminal 21-amino acid signal peptide, three potential *N*-linked glycosylation sites, and two potential *O*-linked glycosylation sites. Multiple sequence alignment with other family GH20 hexosaminidases indicated that the protein sequence of *Mth*NAG has the highest similarity to the GH family 20 protein from *M. thermophila* ATCC 42464 (accession: XP_003658680). The protein sequence of *Mth*NAG shared 80% identity with the GH20 protein from *Thielavia terrestris* NRRL 8126 (accession: XP_003656648) and β-hexosaminidase 2 from *Madurella mycetomatis* (accession: KXX82839), and more than 60% identity was obtained for 79 GH20 proteins from fungal sources including *Fusarium* sp., *Colletotrichum* sp., *Trichoderma* sp., *Scedosporium* sp., and *Neurospora* sp. The protein sequence of *Mth*NAG revealed three domains conserved in family GH-20 hexosaminidases: GH20_HexA_HexB-like domain (accession: cd06562), Glyco_hydro_20 domain (accession: pfam00728), and CHB_HEX domain (accession: PF03173) (Fig. 1c), and a highly conserved pair of catalytic residues D–E preceded by a H-X-G-G motif (Fig. 1a). The modeled secondary structure of *Mth*NAG consists of 16 α-helixes and 21 β-sheets (Fig. 1b), and the modeled tertiary structure has a (α/β)_8_ TIM-barrel structure with the active site lying at the center of the barrel convex side (Fig. 1d).

### Production and purification of MthNAG

**Fig. 2:**
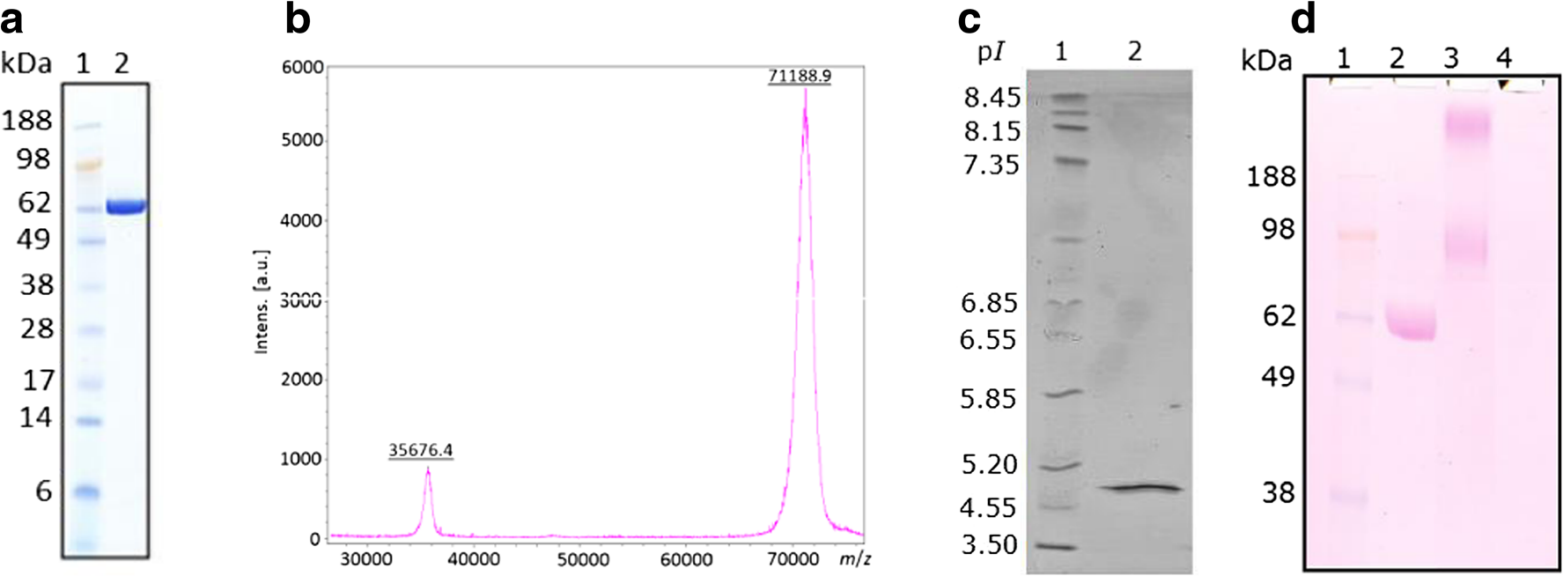
Molecular weight of *Mth*NAG from *Myceliophthora thermophila* C1 determined by **a** SDS-PAGE; *lane 1*: protein marker, *lane 2*: purified *Mth*NAG after size exclusion chromatography (SEC), and molecular weight determined by **b** MALDI-TOF-MS. **c** Isoelectric focusing determined for *Mth*NAG with protein marker (*lane 1*) and purified MthNAG (*lane 2*). **d** Staining with Schiff’s reagent for glycosylated proteins: *lane 1*: protein marker, *lane 2*: purified *Mth*NAG, *lane 3*: yeast invertase, *lane 4*: bovine serum albumin

The gene encoding for *Mth*NAG (*Mthnag)* was amplified using the designed primers and cloned into the *M. thermophila* C1-expression host. The constructed *M. thermophila* C1 strain producing the highest activity of *Mth*NAG was chosen for the production of the enzyme in a 2-L fermenter. Protein concentration in the culture broth was 3.5 g L^−1^, of which the overexpressed *Mth*NAG represented 52% of the total protein. After removal of biomass, the crude enzyme preparation was freeze-dried and further subjected to a purification of *Mth*NAG using IEX and SEC. *Mth*NAG was purified to homogeneity as demonstrated by SDS-PAGE (Fig. 2a) and MALDI-TOF-MS (Fig. 2b). The specific activity of the purified *Mth*NAG was 432 U mg^−1^.

### Molecular weight, isoelectric point, and glycosylation of *Mth*NAG

The molecular weight of *Mth*NAG measured by SDS-PAGE was 62 kDa (Fig. 2a) and that measured by MALDI-TOF-MS was 71.2 kDa (Fig. 2b). In the MALDI-TOF-MS spectrum, the enzyme was identified as a monomeric protein at 71,188.9 [*m*/*z*] (single charged) and at 35,676.4 [*m*/*z*] (double charged). The measured isoelectric point of *Mth*NAG was detected at pH 4.9 (Fig. 2c). Staining of SDS-PAGE gel with PAS resulted in an intense magenta color of the *Mth*NAG protein band (Fig. 2d), showing that the enzyme is glycosylated.

### Biochemical properties of *Mth*NAG

**Fig. 3:**
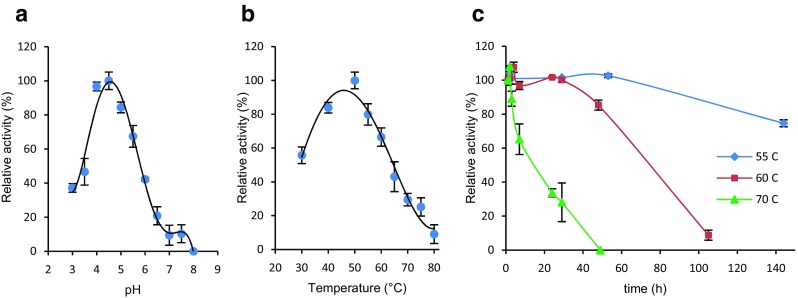
Effects of pH and temperature on the activity of *Mth*NAG from *Myceliophthora thermophila* C1. **a** Optimum pH, **b** optimum temperature, and **c** thermostability

The highest activity of *Mth*NAG was detected at pH 4.5 and 50 °C (Fig. 3 a, b). The activity measurement showed bell-shaped profiles in the pH range of 3 to 8 and between 30 and 80 °C. *Mth*NAG had a relatively high activity (> 40% of the maximum activity) in the range from 30 to 65 °C. At elevated temperatures, above the optimum, *Mth*NAG performed with 30, 25, and 10% of relative activity at 70, 75, and 80 °C, respectively. *Mth*NAG was notably thermostable at 55 °C (75% relative activity, at incubation time > 144 h), 60 °C (85% relative activity, after 48 h incubation; half-life 82 h), and 70 °C (33% relative activity, after 24 h incubation; half-life 18 h) (Fig. 3c).

### GlcNAc and GalNAc release from diverse substrates with *Mth*NAG

The potential of *Mth*NAG to release GlcNAc was examined for the natural chitin dimer (GlcNAc)_2_, chromogenic chitin oligosaccharide derivatives (GlcNAc)_1–3_-*p*NP, and polymeric substrates swollen chitin and soluble chitosan (Table 1). Specific activity for (GlcNAc)_2_ was expressed as the cleavage of two GlcNAc molecules and was 1077.8 ± 0.4 U mg^−1^. Among chitin oligosaccharide *p*NP derivatives, the enzyme showed the highest activity towards GlcNAc-*p*NP, which was about 200-fold higher than that towards (GlcNAc)_2_-*p*NP. Activity towards (GlcNAc)_3_-*p*NP was not detected. *Mth*NAG was able to release GalNAc from GalNAc-*p*NP but with activity lower than that for GlcNAc-*p*NP. Next to oligomeric substrates, *Mth*NAG was active towards polymeric chitin and chitosan. Activity towards chitin and chitosan was about 36,000-fold lower than that obtained for (GlcNAc)_2_. Kinetic parameters for *Mth*NAG were determined for the natural substrate of the enzyme, (GlcNAc)_2_ and its mimic, GlcNAc-*p*NP. For (GlcNAc)_2_, the *V*_max_ was 37.3 ± 5.4 μM min^−1^, *K*_m_ was 0.25 ± 0.05 mM, and *k*_cat_ was 293.7 ± 42.3 s^−1^. For GlcNAc-*p*NP, the *V*_max_ was 1.76 ± 0.09 μM min^−1^, *K*_m_ was 0.06 ± 0.01 mM, and *k*_cat_ was 14.0 ± 0.7 s^−1^. However, *k*_cat_/*K*_m_ for GlcNAc-*p*NP (231.6 s^−1^ mM^−1^) was lower than *k*_cat_/*K*_m_ for (GlcNAc)_2_ (1174.8 s^−1^ mM^−1^). (GlcNAc)_2_ and GlcNAc-*p*NP inhibited the activity of *Mth*NAG, and their inhibition effect was detected at substrate concentrations (*K*_i_) of 0.50 ± 0.11 and 1.96 ± 0.26 mM, respectively.

**Table 1:** Specific activities of *Mth*NAG from *Myceliophthora thermophila* C1

Substrate	Specific activity (U mg^−1^)
(GlcNAc)_2_	1077.8 ± 0.4*
GlcNAc-*p*NP	432.0 ± 0.3**
(GlcNAc)_2_-*p*NP	2.0 ± 0.4**
(GlcNAc)_3_-*p*NP	ND
GalNAc-*p*NP	345.7 ± 3.3**
Swollen chitin	0.03 ± 0.0*
Chitosan (91% DDA)	0.02 ± 0.0***

*ND* not detected

*Measured with high-performance anion-exchange chromatography; **with Tecan Safire; and ***reducing sugar assay

### Release of GlcNAc from chitin oligosaccharides and chitin oligosaccharide *p*NP derivatives with *Mth*NAG

**Fig. 4:**
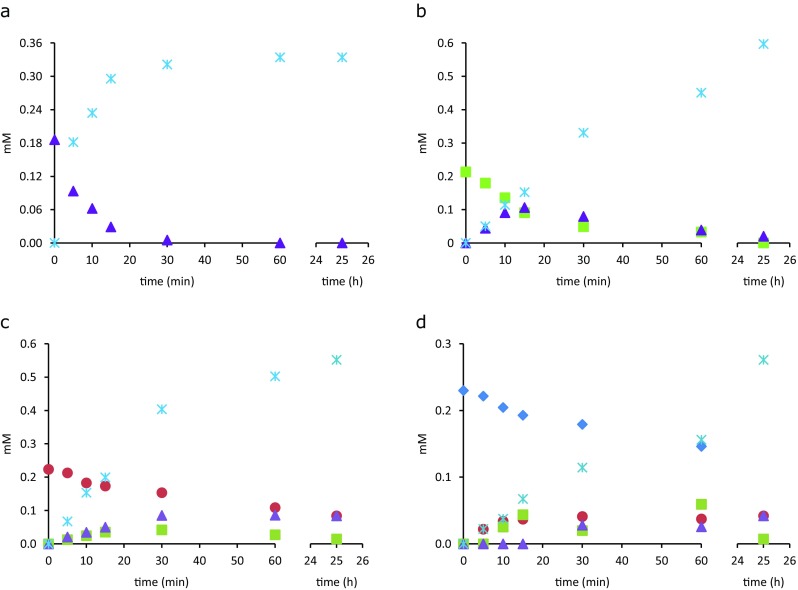
Hydrolysis of chitin oligosaccharides (GlcNAc)_2–5_ by *Mth*NAG from *Myceliophthora thermophila* C1. Reaction products obtained after incubation of *Mth*NAG with (GlcNAc)_2_ (**a**), (GlcNAc)_3_ (**b**), (GlcNAc)_4_ (**c**), and (GlcNAc)_5_ (**d**), identified by high-performance anion-exchange chromatography (HPAEC). GlcNAc, star; (GlcNAc)_2_, triangle; (GlcNAc)_3_, square; (GlcNAc)_4_, circle; (GlcNAc)_5_, diamond

**Fig. 5:**
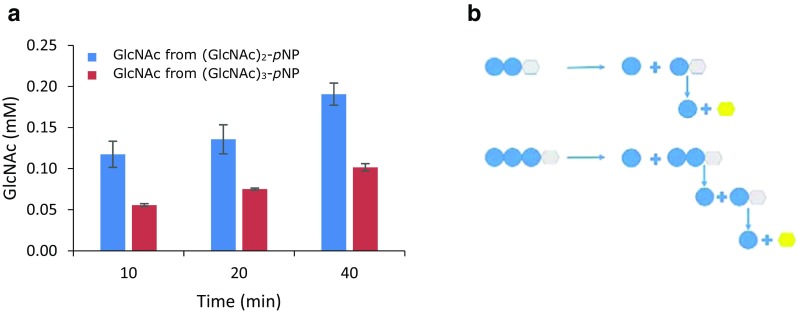
Cleavage of (GlcNAc)_2_-*p*NP and (GlcNAc)_3_-*p*NP with *Mth*NAG from *Myceliophthora thermophila* C1. **a** Time course for degradation of (GlcNAc)_2_-*p*NP and (GlcNAc)_3_-*p*NP and **b** mode of action of *Mth*NAG. GlcNAc depicted as blue circles and *p*-nitrophenol (*p*NP) as hexagons. Yellow color indicates ionization of released *p*NP

The release of GlcNAc from chitin oligosaccharides (GlcNAc)_2–5_ catalyzed by *Mth*NAG was followed in time, and the reaction products were measured by HPAEC (Fig. 4). *Mth*NAG efficiently degraded (GlcNAc)_2_ to two molecules of GlcNAc, and already after 30 min, the substrate was fully degraded (Fig. 4a). All other oligosaccharides (GlcNAc)_3–5_ were shortened by one GlcNAc molecule in time, and the released intermediate products (GlcNAc)_*n* − 1_ were simultaneously degraded during the reaction. In the first 15 min, (GlcNAc)_3_ was degraded to molar equivalent concentration of GlcNAc and (GlcNAc)_2_ (Fig. 4b), and the released (GlcNAc)_2_ dimers were degraded gradually hereafter. Hydrolysis of (GlcNAc)_4_ and (GlcNAc)_5_ resulted in the release of GlcNAc and the intermediate (GlcNAc)_*n* − 1_ oligosaccharides, which were further degraded (Fig. 4c, d). The degradation rates in the first 5 min were as follows: 18.5 μM/min for (GlcNAc)_2_, 6.6 μM/min for (GlcNAc)_3_, 2.2 μM/min for (GlcNAc)_4_, and 1.7 μM/min for (GlcNAc)_5_. To determine the mode of action of *Mth*NAG, the enzyme was incubated with (GlcNAc)_2–3_-*p*NP and the release of the products was followed in time. As shown in Fig. 5, degradation of (GlcNAc)_2–3_-*p*NP progressed with the release of GlcNAc and intermediate (GlcNAc)_*n* − 1_-*p*NP, which were subsequently hydrolyzed by the enzyme. Similarly to chitin oligosaccharides, the enzyme showed preference towards shorter oligosaccharide substrate, since it degraded (GlcNAc)_2_-*p*NP two times faster than (GlcNAc)_3_-*p*NP (Fig. 5a). GlcNAc was cleaved by the enzyme from the non-reducing end of the substrate (Fig. 5b).

### Effect of *Mth*NAG and Chitinase Chi1 on the release of GlcNAc from swollen chitin

Chitinase Chi1 from *M. thermophila* C1 was previously shown to release mainly (GlcNAc)_2_ and traces of (GlcNAc)_3_ and GlcNAc from swollen chitin (Krolicka et al. 2018). In this work, Chitinase Chi1 and *Mth*NAG were used to examine their synergistic effect on the release of GlcNAc from swollen chitin by performing two parallel runs and measuring the concentration of released GlcNAc in time. In run I, containing only *Mth*NAG, the concentration of the released GlcNAc at the end of the reaction was 3.1 × 10^−3^ mM, while in run II, containing both enzymes, the concentration of GlcNAc was 0.39 mM at the end of incubation (Table 2). GlcNAc yield obtained with the mixture of both enzymes was equal to 37.8% which is about 13 times higher when only *Mth*NAG was used (2.9%).

**Table 2:** Release of GlcNAc during the hydrolysis of swollen chitin by *Mth*NAG (run I) and by the action of both *Mth*NAG and Chitinase Chi1 (run II) from *Myceliophthora thermophila* C1

Time (min)	Run I (*Mth*NAG)		Run II (*Mth*NAG + chitinase Chi 1)	
	GlcNAc concentration (mM)	Glcnac yield (%)	GlcNAc concentration (mM)	GLCNAC yield (%)
15	0.443 × 10^−3^	0.4	0.12	4.9
30	0.488 × 10^−3^	0.5	0.14	5.9
60	0.815 × 10^−3^	0.8	0.19	7.9
90	1.014 × 10^−3^	0.9	0.22	9.0
120	1.184 × 10^−3^	1.2	0.24	9.7
1140	3.078 × 10^−3^	2.9	0.39	37.8

## Discussion

The multiple sequence alignment of the protein sequence of *Mth*NAG revealed the presence of conserved domains and catalytic residues typically found in GH20 family hexosaminidases. The GH20_HexA_HexB-like domain is a representative domain of the active site of GH20 hexosaminidases from microorganisms and higher organisms. The CHB_HEX superfamily domain was suggested to be a carbohydrate binding domain since it resembles the crystallographic structure of a cellulose binding domain in cellulase from *Cellulomonas fimi* (Xu et al. 1995; Tews et al. 1996). Putative features for protein maturation were found in *Mth*NAG, including a 21-amino acid signal peptide and five glycosylation motifs. Tertiary structure predicted with 3D modeling revealed an (α/β)_8_ TIM-barrel conformation of *Mth*NAG, which is typical for GH20 hexosaminidases. According to all these features, *Mth*NAG can be classified to GH20 hexosaminidases.

The molecular weight of *Mth*NAG measured with SDS-PAGE is in the range of molecular weights measured with SDS-PAGE for other fungal NAGases (Table 3). However, the molecular weight of *Mth*NAG measured with MALDI-TOF-MS (71.2 kDa) was 8.6 kDa higher than the molecular weight calculated from the protein sequence (62.6 kDa). This difference is most likely due to the post-translational glycosylation of *Mth*NAG performed by *M. thermophila* C1, as the purified *Mth*NAG gave a strong magenta signal upon Schiff’s staining. Carbohydrates could potentially attach to the five glycosylation sites predicted in the *Mth*NAG sequence. A similar increase in molecular weight of about 10 kDa caused by glycosylation was observed for NAGase from *T*. *harzianum* P1 (62.7 kDa) (Peterbauer et al. 1996). However, *Mth*NAG and NAGase from *T*. *harzianum* P1 migrated differently in the SDS-PAGE. *Mth*NAG appeared as a protein with lower molecular weight (62 kDa), while NAGase from *T*. *harzianum* P1 appeared as a protein with higher molecular weight (72 kDa). Anomalous behavior of glycoproteins has been described before, and the presence of carbohydrates attached to the protein (Segrest et al. 1971; Matagne et al. 1991) and the tertiary structure of the protein (Rath et al. 2009; Pitt-Rivers and Ambesi Impiombato 1968) were shown to influence this behavior. MALDI-TOF-MS is known to provide accurate molecular mass determination of proteins and glycoconjugates (Ledesma-Osuna et al. 2008; Yeboah and Yaylayan 2001).

**Table 3:** Properties of hexosaminidases from fungi

Organism	Molecular weight (kDa)	Optimum pH	Optimum temperature (°C)	*K*_m_ (mM)	p*I*	Activity (U mL^−1^)	Specific activity (U mg^−1^)	Substrate diversity	Reference
*Aspergillus nidulans*	190	5.0	50	0.18*, 0.58**	4.3	28**	47**	GlcNAc-*p*NP, GalNAc-*p*NP, GlcNAc-4-MUF, GalNAc-4-MUF, (GlcNAc)_2–6_	Reyes et al. 1989
*Aspergillus nidulans*	65	4.0–5.0	52	–	–	–	–	GlcNAc-*p*NP, GalNAc-*p*NP	Kim et al. 2002
*Aspergillus niger* 419	131	4.5	65	0.2**	4.4	13.5**	71**	GlcNAc-*p*NP, GalNAc-*p*NP, (GlcNAc)_2_-*p*NP	Pera et al. 1997
*Aspergillus niger*	149	2.95–8.25	–	0.34**, 0.86***	4.4	3261**	72.2**, 44.6***	GlcNAc-*p*NP, GalNAc-*p*NP, GlcN-TAc-*p*NP, GlcNAc-2,4-di*p*NP	Jones and Kosman 1980
*Aspergillus niger*	–	3.9–4.6	–	0.66**	–	610**	7170**	(GlcNAc)_2–6_, glycopeptides derived from ovalbumin, β-galactosidase-treated desialyzed fetuin, α1-acid glycoprotein	Bahl and Agrawal 1969
*Aspergillus oryzae*	65	5.0	–	–	–	1926**	1926**	GlcNAc-*p*NP, GalNAc-*p*NP, synthesis of lacto-*N*-triose from GlcNAc and lactose	Matsuo et al. 2003
*Aspergillus oryzae*	140	4.5	–	0.85**	–	10,400**	242**	GlcNAc-*p*NP, GalNAc-P, GlcNAc-P	Mega et al. 1970
*Beauveria bassiana*	97 (NAGase 1) 130 (NAGase 2)	5.0 5.0	57 37	0.38** 2.10**	9.5 5.5	5175** 28,040**	3080** 14,758**	GlcNAc-*p*NP	Bidochka et al. 1993
*Candida albicans*	64	–	–	–	–	274**	288**	GlcNAc-*p*NP	Cannon et al. 1994
*Candida albicans*	44	6.5	–	0.45**	–	1.4**	36.8**	GlcNAc-*p*NP, (GlcNAc)_2–3_	Sullivan et al. 1984
*Fusarim oxysporum F3*	67 (NAG I) 67 (NAG II)	5.0 6.0	45 45	0.05** 0.05**	– –	14.1** 9.0**	176.2** 100**	GlcNAc-*p*NP	Gkargkas et al. 2004
*Lentinula edodes*	79	4.0	50	0.42*, 0.34**	–	– –	46.3¥, 39.9§	GlcNAc-*p*NP, GalNAc-*p*NP, (GlcNAc)_2–6_, colloidal chitin, mechanochemically ground chitin	Konno et al. 2012
*Metarhizium anisopliae*	110	5.0	–	–	6.4	–	2.65†, 1.21#	GlcNAc-*p*NP, GalNAc-*p*NP, (GlcNAc)_2–4_, colloidal chitin	St. Leger et al. 1991
*Neotyphodium* sp.	60	–	–	–	–	–	–	GlcNAc-*p*NP	Li et al. 2005
*Paecilomyces persicinus*	95	4.6	35–37	0.63**	4.8	46.3**, 28.2***	9.26**, 5.64***	GlcNAc-*p*NP, GalNAc-*p*NP	Eriquez and Pisano 1979
*Penicillium chrysogenum*	66.5	–	–	–	–	–	–	–	Diez et al. 2005
*Penicillium oxalicum*	141	3–4.5	–	0.48**	5.0	1660** 2450***	126** 186***	GlcNAc-*p*NP, GalNAc-*p*NP	Yamamoto et al. 1985
*Penicillium oxalicum*	160	3.0	50	0.14**	–	–	10.8**	GlcNAc-*p*NP, GalNAc-*p*NP, GlcNAc-4-MUF	Ryslava et al. 2011
*Phoma glomerata*	20	6.0–8.0	37	–	–	38.2**	1020**	GlcNAc-4-MUF, GalNAc-4-MUF	Zhuravleva et al. 2004
*Pycnoporus cinnabarinus*	120	2.2	–	–	5.4	134** 149***	67** 75***	GlcNAc-*p*NP, GalNAc-*p*NP, (GlcNAc)_2–4_	Ohtakara et al. 1981
*Trichoderma harzianum* P1	72	5.0–5.5	60	–	4.6	17.4‡	11.8‡	(GlcNAc)_1–2_-*p*NP, Fungal cell walls	Lorito et al. 1994
*Trichoderma harzianum*	36	4.0	50–60	0.008**	–	–	–	GlcNAc-*p*NP, fungal cell walls	Lisboa De Marco et al. 2004
*Trichoderma harzianum*	118	5.5	50	0.24*, 0.58**	–	7.3**	73**	GlcNAc-*p*NP	Ulhoa and Peberdy 1991
*Trichoderma harzianum* AF 6-T8	150	5.2	50	–	–	960**	102**	GlcNAc-*p*NP, (GlcNAc)_2–6_	Koga et al. 1991
*Myceliophthora thermophila* C1	71.2	4.5	50	0.25*, 0.06 **	4.9	1357* 544**	1077.8* 432**	GlcNAc-*p*NP, GalNAc-*p*NP, (GlcNAc)_2–3_-*p*NP, (GlcNAc)_2–6_, swollen chitin, chitosan 91% DDA	This paper

*For (GlcNAc)_2_; **for GlcNAc-*p*NP; ***for GalNAc-*p*NP; ¥for colloidal chitin; §for mechanochemically ground chitin; †for (GlcNAc)_2_ in micromoles per hour; #for GlcNAc-*p*NP in micromoles per hour; ‡for GlcNAc-*p*NP in nanokatals

*Mth*NAG was found to have a pH optimum at pH 4.5 and a p*I* of 4.9, while the calculated p*I* from the amino acid sequence was 5.2. This difference between the theoretical p*I* predicted from the primary structure and the experimentally determined p*I* is common, and is in the error limits of the method (Kozlowski 2016).

*Mth*NAG exhibits the highest catalytic activity at 50 °C, similar to other thermophilic fungal GH20 hexosaminidases (Table 3). Interestingly, *Mth*NAG showed a notable thermostability above its optimal temperature, i.e., at 55, 60, and 70 °C, that to our knowledge has never been observed for other fungal NAGases. Literature reported fungal thermophilic NAGases with temperature optimum ≥ 50 °C which drastically lose their activities after prolonged incubation at optimal or higher temperatures. For example, NAGase 1 from *B. bassiana* has its temperature optimum at 57 °C but it lost 100% of activity after 60 min incubation at that temperature (Bidochka et al. 1993). NAGase from *T. harzainum* with an optimum at 50–60 °C lost 50% of its original activity within 1 h at 50 °C (Lisboa De Marco et al. 2004). At elevated temperatures, *Mth*NAG was more stable than LeHex20A from *L. edodes* and NAGase from *A. niger* 419. At 60 °C, *Mth*NAG retained 85% relative activity for 48 h, while LeHex20A from *L. edodes* was inactivated within 30 min (Konno et al. 2012). NAGase from *A. niger* 419, which was most active at 65 °C, lost 30% of activity after 15 min at 70 °C (Pera et al. 1997), while *Mth*NAG lost 35% of activity after 5 h at that temperature. The notable thermostability of *Mth*NAG is probably an evolutionary adaptation to preserve enzymatic function at elevated temperatures. This adaptation may include stabilizing interactions in folded protein, interactions between domains and presence of stable surface-exposed amino acids (Turner et al. 2007). Glycosylation of *Mth*NAG may have also a positive effect on the thermostability, as glycosylation was shown to stabilize the enzyme conformation of glucoamylase from *A. niger* (Jafari-Aghdam et al. 2005) and improved the pH stability of NAGase PoHEX from *Penicillium oxalicum* (Ryslava et al. 2011).

*Mth*NAG was very active towards the dimeric substrate (GlcNAc)_2_ and its mimic GlcNAc-*p*NP, which are typical substrates for *N*-acetylglucosaminidases. In addition, *Mth*NAG showed *N*-acetylgalactosaminidase activity as it released the GalNAc moiety from GalNAc-*p*NP. The activity ratio of 1.25 between *N*-acetylglucosaminidase and *N*-acetylgalactosaminidase activities indicates that the enzyme has more or less the same activity towards GalNAc and GlcNAc conjugates. *N*-Acetylglucosaminidase and *N*-acetylgalactosaminidase activity are commonly found for members of the GH20 hexosaminidase family.

*Mth*NAG showed activity for the trimeric chitin oligosaccharide derivative (GlcNAc)_2_-*p*NP, but no activity was detected towards the tetrameric (GlcNAc)_3_-*p*NP (Table 1). However, a time-course experiment with (GlcNAc)_2–3_-*p*NP revealed that GlcNAc was released from both substrates (Fig. 5) and that *Mth*NAG released GlcNAc from the non-reducing end of the substrate. In the case of enzymes acting from the non-reducing end, the release of *p*NP from chitin oligosaccharide *p*NP derivatives depends on the length of the *p*NP derivative and the longer the *p*NP derivative, the more time the enzyme needs to reach the attached *p*NP and to release the dye. Therefore, it should be noted that the spectrophotometric activity measurement with *p*NP derivatives may give false results when the mode of action of the enzyme is not taken into account.

Studies of the kinetic parameters *Mth*NAG indicated that (GlcNAc)_2_ is a good substrate for the enzyme, but the enzyme has a lower affinity for the natural substrate (higher *K*_m_) than for the synthetic mimic GlcNAc-*p*NP (lower *K*_m_). However, the higher catalytic efficiency (*k*_cat_ and *k*_cat_/*K*_m_) towards (GlcNAc)_2_ indicates that (GlcNAc)_2_ is a more preferred substrate than GlcNAc-*p*NP. The *K*_m_ for (GlcNAc)_2_ was comparable with the one from LeHex20A from *L. edodes* (Konno et al. 2012), although it was higher than the reported values for NAGase from *T. harzianum* (Ulhoa and Peberdy 1991) (Table 3). Although, *Mth*NAG showed high enzymatic activity towards (GlcNAc)_2_ and GlcNAc-*p*NP, it was also inhibited by these substrates. The measured *K*_i_ for (GlcNAc)_2_ was equal to two times of *K*_m_, indicating that at *K*_i_ enzyme works at its *V*_max_. For setting a process, it is important to work below the *K*_i_ for (GlcNAc)_2_, which can be achieved by slowly adding the substrate. Substrate inhibition is also reported for other NAGases. For example, GlcNAc-*p*NP at a concentration of 0.4 mM inhibited fungal NAGase PoHEX from *P. oxalicum* (Ryslava et al. 2011). Blind docking experiment conducted on PoHEX revealed that substrate inhibition was a result of the presence of a “secondary” binding site. Whether such an additional binding site is a reason of substrate inhibition by *Mth*NAG, additional blind docking experiment should be conducted for *Mth*NAG.

Next to hydrolysis of (GlcNAc)_2_, *Mth*NAG was capable of cleaving off GlcNAc moieties from chitin oligosaccharides (GlcNAc)_3–5_. The activity on chitin oligosaccharides has been reported for some other NAGasses (Table 3). The enzyme showed the highest preference towards (GlcNAc)_2_. In the first 5 min of incubation, (GlcNAc)_2_ was converted approximately three times faster than (GlcNAc)_3_ and almost 10 times faster than (GlcNAc)_4_ and (GlcNAc)_5_. *Mth*NAG degraded chitin oligosaccharides (GlcNAc)_3–5_ non-processively in a consecutive reaction, which means that oligosaccharides were shortened by GlcNAc in time and their intermediate (GlcNAc)_*n* − 1_ were simultaneously degraded by the enzyme. In this way, the inhibition concentration of (GlcNAc)_2_ is not reached and the reaction can proceed to completion.

Fungal NAGases differ in their substrate diversity and activity towards GlcNAc-*p*NP (Table 3). They are in general known to degrade chitin dimers and oligosaccharides, but activity on polymeric chitin is not common for this group of enzymes. Activity towards chitin and chitosan makes *Mth*NAG a unique enzyme among NAGases. Only a few bacterial NAGases and the fungal LeHex20A from *L. edodes* and *M. anisopliae* were reported to degrade chitin to some extent (Suginta et al. 2010; Konno et al. 2012; St. Leger et al. 1991). Activity on chitosan has not been reported before. Keeping in mind that *Mth*NAG releases a single GlcNAc moiety from the non-reducing end, depolymerization of chitosan is only possible through the release of GlcNAc from the terminal site of chitosan chains. Furthermore, this result implies the acetylation profile of the chitosan used in the experiment (90% DDA), in which chitosan has numerous acetylated moieties at its non-reducing ends.

The activity on polymeric substrates may indicate that *Mth*NAG could release GlcNAc moiety from glycopeptides, as it was reported for NAGase from *A. niger* (Bahl and Agrawal 1969).

However, the activity of *Mth*NAG towards chitin and chitosan is relatively low and potential industrial production of GlcNAc from chitin cannot be based only on this enzyme. Therefore, chitin depolymerization was tested with addition of Chitinase Chi1, which was shown to release mainly (GlcNAc)_2_ from chitin (Krolicka et al. 2018). *Mth*NAG and Chitinase Chi1 were expected to work in synergy, i.e., the (GlcNAc)_2_ released from chitin by Chitinase Chi1 will be hydrolyzed by *Mth*NAG to GlcNAc. The amount of GlcNAc released by *Mth*NAG and Chitinase Chi1 was compared with the amount released by *Mth*NAG only. It was observed that when the enzymes worked together, they were able to release about 130 times more GlcNAc from swollen chitin than when only *Mth*NAG was used. This result strongly indicates that these enzymes indeed work in synergy and can be used for the setup of an enzymatic production process of GlcNAc. The high thermostability of Chitinase Chi1 and *Mth*NAG is an additional advantage for their potential industrial application.

In conclusion, a novel β-*N*-acetylglucosaminidase from the thermophilic fungus *M. thermophila* C1 was cloned and homologously overexpressed. The enzyme showed high specific activity towards (GlcNAc)_2_. *Mth*NAG is notably thermostable, and to the best of our knowledge, *Mth*NAG is the first reported fungal NAGase with such high thermostability. Together with Chitinase Chi1, *Mth*NAG released about 130 times more GlcNAc from swollen chitin than when used alone. Application of both *Mth*NAG and Chitinase Chi1 as a two-enzyme catalyst is a promising tool for production of GlcNAc from chitin. Furthermore, *Mth*NAG can be used for determination of the deacetylation pattern of chitosan. In addition, due to the wide substrate specificity of *Mth*NAG, this enzyme has potential to be used in combinations with other enzymes for deglycosylation of glycoconjugates such as glycans, glycoproteins, and glycolipids and for elucidation of their structure or changing their biological activity.
